# Editorial: National Cancer Research Month 2025: Advances in Detection, Treatment and Therapies in Oncology

**DOI:** 10.3389/fonc.2026.1834671

**Published:** 2026-04-20

**Authors:** James C. L. Chow, Daniel P. Bezerra, Valdir C. Colussi, Matiullah Khan, Cecilia A. Suárez, Tamer S. Kaoud

**Affiliations:** 1 Department of Radiation Oncology, University of Toronto, Toronto, ON, Canada; 2 Radiation Medicine Program, Princess Margaret Cancer Centre, University Health Network, Toronto, ON, Canada; 3 Gonçalo Moniz Institute, Oswaldo Cruz Foundation (IGM-FIOCRUZ/BA), Salvador, Bahia, Brazil; 4 Department of Radiation Oncology, Case Western Reserve University, Cleveland, OH, United States; 5 Seidman Cancer Center, University Hospitals (UH) Cleveland, Cleveland, OH, United States; 6 Department of Pathology, Faculty of Medicine, AIMST University, Bedong, Malaysia; 7 Physics Department, University of Buenos Aires, Buenos Aires, Argentina; 8 Interdisciplinary and Applied Physics Institute, University of Buenos Aires - National Scientific and Technical Research Council, Buenos Aires, Argentina; 9 Department of Pharmaceutical Sciences, College of Pharmacy, University of Arkansas for Medical Sciences, Little Rock, AR, United States; 10 Winthrop P. Rockefeller Cancer Institute, University of Arkansas for Medical Sciences, Little Rock, AR, United States

**Keywords:** cancer detection, genomic profiling, machine learning in oncology, molecular mechanisms, precision oncology, radiotherapy advances, rare cancers, translational cancer research

## Introduction

Cancer remains one of the leading causes of morbidity and mortality worldwide, demanding continuous innovation in biological understanding, technological development, and clinical practice. Progress in oncology increasingly depends on the integration of fundamental discoveries with translational research and data-driven clinical decision-making. The eighteen contributions assembled in this Research Topic Collection for National Cancer Research Month 2025 reflect this integrative momentum, spanning molecular mechanisms foundations of tumor biology advances in radiotherapy and computational science, innovations in surgical and locoregional management, and insights from population genomics and rare tumor studies. Together, these studies illustrate how multidisciplinary research continues to shape a more precise and biologically informed approach to cancer care.

## Biological mechanisms and molecular insights

Understanding how tumors arise, adapt, and evade therapy remains central to modern oncology. Conceptual frameworks such as the Hallmarks of Cancer, introduced by Douglas Hanahan and Robert A. Weinberg and recently revisited by Douglas Hanahan, provide a unifying perspective for interpreting the biological processes that drive tumorigenesis and therapeutic resistance (1–3). Several contributions in this Research Topic build on this conceptual foundation to deepen the mechanistic understanding of cancer development and treatment response.

Brahme presents a comprehensive review of quantum scale radiation interactions describing how low-energy delta rays can produce concentrated DNA damage that influences biological effectiveness and informs future optimization of fractionation strategies and linear energy transfer selection in clinical radiotherapy. Complementing this perspective, Liu et al. explore how RNA methylation marks such as m6A, m5C, and m7G regulate gene expression and contribute to radioresistance, outlining potential strategies for targeting these pathways to increase therapeutic sensitivity. Hormone signaling and receptor conformation also feature prominently in this Research Topic. Saha and Lukong provide a detailed structural and functional overview of ERα signaling, ESR1 mutations, and GPER activity in endocrine-resistant breast cancer and highlight the need for therapeutic strategies that address both nuclear and membrane mediated signaling. Moreover, Ji et al. expanded the biomarker landscape by reviewing claudin family proteins, describing their roles as modulators of cell adhesion, invasion, and metastasis, and discussing the emergence of claudin targeted therapies across multiple cancer types.

Other mechanistic work focuses on pathways not traditionally centered in oncology. Meyer et al. examine the role of the coagulation factors FXa and PAR2 in colon cancer progression and reveal how FXa signaling enhances tumor proliferation and migration, providing a novel axis for potential intervention. Akhtar et al. study several microRNAs in leiomyosarcoma and confirm their upregulation in tumor tissue while noting their limited predictive value for metastatic risk, which is important for future biomarker validation. In addition, Ma et al. address a long standing clinical problem by reviewing high risk contributors to radioiodine refractoriness in differentiated thyroid cancer. Their analysis includes demographic factors, genetic mutations such as BRAF and RAS, histopathological patterns, serum markers, and comorbidity related influences on iodine uptake. These papers collectively show how a deeper molecular understanding provides the foundation for more precisely targeted and biologically informed therapies.

## Technological innovation, clinical translation, and decision support

Technological innovation continues to reshape oncology by translating biological knowledge into improved diagnostic and therapeutic approaches. Large-scale cancer genomics initiatives such as The Cancer Genome Atlas and the International Cancer Genome Consortium have revealed the genomic heterogeneity of tumors and identified actionable molecular alterations across numerous cancer types (4–6). These advances underpin the emergence of precision oncology and increasingly data-driven clinical decision-making.

Several contributions highlight how evolving technologies and analytical methods are reshaping treatment design and clinical decision making. Amin et al. analyze survival outcomes in more than 117,000 sarcoma patients in the United States National Cancer Database and report that compared with photon therapy, proton beam therapy is associated with improved overall survival in several clinically important histologies. Sun et al. review international experience with boron neutron capture therapy for melanoma and outline recent improvements in accelerator systems and boron delivery agents that support continued development. On the other hand, Aigner et al. introduce a regional treatment approach for anal squamous cell carcinoma that uses isolated hypoxic pelvic perfusion with or without reversible electroporation. Their reported cases show complete clinical and pathological remissions with minimal toxicity, demonstrating a promising alternative for select patients.

Precision oncology also benefits from advances in molecular profiling. Erices et al. produce an ancestry informed genomic map of gallbladder cancer in Chile, identifying actionable alterations in genes including ATM, BRCA1/2, EGFR, and ERBB2. They also report an association between Mapuche ancestry and TP53 mutations, underscoring the value of population specific genomic evidence. Hofmann et al. present a case of colorectal cancer with an ALK CEP44 fusion that initially responds to ALK inhibition before the rapid development of multiple resistance mutations, illustrating the dynamic nature of tumor evolution and the need for serial molecular assessment.

Machine learning is represented by Antysheva et al. who develop a single sample molecular classifier for cancer grading that operates without batch correction and provides reliable risk stratification for intermediate grade tumors, an important step toward real world applicability. Important insights into surgical practice and outcome prediction are also included in this group. Yumak and Demir report that lymphovascular invasion, perineural invasion, poor differentiation, R1 resection, emergency surgery, and subsequent metastasis are dominant predictors of mortality in patients with rectal cancer. Furthermore, Lin et al. evaluate risk factors for contralateral central lymph node metastasis in papillary thyroid cancer and show that microcalcification and Hashimoto thyroiditis are strong predictors that support selective contralateral dissection. These papers collectively demonstrate how technologies such as particle therapy, machine learning, advanced profiling, and carefully analyzed clinical data are transforming how clinicians tailor treatment for specific patients and settings.

## Rare cancers, clinical insight, and conceptual framing

Rare cancers collectively represent a substantial yet often underrecognized portion of the global cancer burden, accounting for approximately 20–25% of malignancies (7, 8). Their study provides unique opportunities to uncover novel biological mechanisms and refine clinical management strategies through collaborative research networks and shared data resources.

In this Research Topic, several case-based contributions illustrate the diagnostic and therapeutic challenges posed by uncommon malignancies. Yu et al. report a case of recurrent mucinous adenocarcinoma of the prostate, emphasizing the diagnostic reliance on pathology and the multimodal treatment required for this very uncommon disease. Li et al. present a case of breast carcinosarcoma and highlight the challenges of diagnosing tumors with nonspecific clinical findings, reinforcing the importance of comprehensive pathology and coordinated therapy. Finally, Bredberg offers a broader perspective on cancer complexity and argues that cancer should be viewed as a complex adaptive system that requires conceptual tools from complexity science in addition to traditional target based strategies. His commentary encourages the field to recognize the nonlinear and multiscale behavior inherent to cancer progression.

## Conclusion

Together, these eighteen studies presented in this Research Topic reflect the dynamic and multidisciplinary nature of contemporary cancer research. They illustrate progress across molecular biology, precision radiotherapy, genomic profiling, machine learning, surgical decision-making, and the clinical understanding of rare cancers. More broadly, they highlight how progress in cancer research increasingly emerges from the integration of biological insight, technological innovation, and clinical expertise. Continued collaboration across disciplines will be essential to translate these advances into more effective, personalized, and equitable cancer care.
