# Correction: Editorial: National Cancer Research Month 2025: Advances in Detection, Treatment and Therapies in Oncology

**DOI:** 10.3389/fonc.2026.1867092

**Published:** 2026-05-15

**Authors:** James C. L. Chow, Daniel P. Bezerra, Valdir C. Colussi, Matiullah Khan, Cecilia A. Suárez, Tamer S. Kaoud

**Affiliations:** 1 Department of Radiation Oncology, University of Toronto, Toronto, ON, Canada; 2 Radiation Medicine Program, Princess Margaret Cancer Centre, University Health Network, Toronto, ON, Canada; 3 Gonçalo Moniz Institute, Oswaldo Cruz Foundation (IGM-FIOCRUZ/BA), Salvador, Bahia, Brazil; 4 Department of Radiation Oncology, Case Western Reserve University, Cleveland, OH, United States; 5 Seidman Cancer Center, University Hospitals (UH) Cleveland, Cleveland, OH, United States; 6 Department of Pathology, Faculty of Medicine, AIMST University, Bedong, Malaysia; 7 Physics Department, University of Buenos Aires, Buenos Aires, Argentina; 8 Interdisciplinary and Applied Physics Institute, University of Buenos Aires - National Scientific and Technical Research Council, Buenos Aires, Argentina; 9 Department of Pharmaceutical Sciences, College of Pharmacy, University of Arkansas for Medical Sciences, Little Rock, AR, United States; 10 Winthrop P. Rockefeller Cancer Institute, University of Arkansas for Medical Sciences, Little Rock, AR, United States

**Keywords:** cancer detection, genomic profiling, machine learning in oncology, molecular mechanisms, precision oncology, radiotherapy advances, rare cancers, translational cancer research

## Incorrect funding

An incorrect **Funding** statement was provided. In the published article, there was a mistake in the **Funding** statement. The funding statement was displayed as “The author(s) declared that financial support was received for this work and/or its publication. TSK was supported by the National Institute of General Medical Sciences of the National Institutes of Health, Award Number P20GM152281. The content is solely the responsibility of the authors and does not necessarily represent the official views of the National Institutes of Health.”

The correct **Funding** statement is:

The author(s) declare that no financial support was received for this work and/or its publication.

The original version of this article has been updated.

